# Epigenetic Changes during Hepatic Stellate Cell Activation

**DOI:** 10.1371/journal.pone.0128745

**Published:** 2015-06-12

**Authors:** Silke Götze, Eva C. Schumacher, Claus Kordes, Dieter Häussinger

**Affiliations:** Clinic of Gastroenterology, Hepatology and Infectious Diseases, Heinrich Heine University, Düsseldorf, Germany; University of Navarra, SPAIN

## Abstract

**Background and Aims:**

Hepatic stellate cells (HSC), which can participate in liver regeneration and fibrogenesis, have recently been identified as liver-resident mesenchymal stem cells. During their activation HSC adopt a myofibroblast-like phenotype accompanied by profound changes in the gene expression profile. DNA methylation changes at single genes have been reported during HSC activation and may participate in the regulation of this process, but comprehensive DNA methylation analyses are still missing. The aim of the present study was to elucidate the role of DNA methylation during *in vitro* activation of HSC.

**Methods and Results:**

The analysis of DNA methylation changes by antibody-based assays revealed a strong decrease in the global DNA methylation level during culture-induced activation of HSC. To identify genes which may be regulated by DNA methylation, we performed a genome-wide Methyl-MiniSeq EpiQuest sequencing comparing quiescent and early culture-activated HSC. Approximately 400 differentially methylated regions with a methylation change of at least 20% were identified, showing either hypo- or hypermethylation during activation. Further analysis of selected genes for DNA methylation and expression were performed revealing a good correlation between DNA methylation changes and gene expression. Furthermore, global DNA demethylation during HSC activation was investigated by 5-bromo-2-deoxyuridine assay and L-mimosine treatment showing that demethylation was independent of DNA synthesis and thereby excluding a passive DNA demethylation mechanism.

**Conclusions:**

In summary, *in vitro* activation of HSC initiated strong DNA methylation changes, which were associated with gene regulation. These results indicate that epigenetic mechanisms are important for the control of early HSC activation. Furthermore, the data show that global DNA demethylation during activation is based on an active DNA demethylation mechanism.

## Introduction

Hepatic stellate cells (HSC) have recently been identified as liver-resident mesenchymal stem cells and are thought to contribute to liver repair and fibrogenesis [1–3]. In the liver, HSC reside between sinusoidal endothelial cells and hepatocytes in the space of Disse, which serves as a stem cell niche for stellate cells [4,5]. Quiescent HSC are characterized by high content of vitamin A mainly stored as retinyl-palmitate in membrane-coated lipid droplets and expression of filamentous proteins like glial fibrillary acidic protein (Gfap) and desmin [6,7]. During activation HSC lose their vitamin A stores and develop into myofibroblast-like cells. Activated HSC start to express α-smooth muscle actin (αSma) and extracellular matrix proteins such as collagen type I, a process involved in liver fibrosis. Despite their role during fibrogenesis the true function of HSC in the normal, uninjured liver remained unknown. Recently, HSC have been described as mesenchymal stem cells due to their potential to differentiate into adipocytes and osteocytes and their ability to support hematopoiesis [1,8]. Furthermore, HSC are important players during liver regeneration, where they can either support regeneration through the secretion of mitogenic factors like hepatocyte growth factor (Hgf) [9] or even participate directly in regeneration by differentiating into hepatocytes as shown in a stem-cell based liver regeneration model in rat [2].

The term epigenetics summarizes all heritable changes of gene expression that occur without alterations of the DNA sequence. Different epigenetic mechanisms are known, which can regulate the gene expression like DNA methylation, histone modifications and miRNAs [10]. DNA methylation is performed by DNA methyltransferases (Dnmt), which transfer a methyl group from an S-adenosylmethionine (SAM) to a cytosine in a CpG-dinucleotide (cytosine-phosphate-guanine) sequence. The influence of DNA methylation on gene expression strongly depends on the genomic context. While DNA methylation at promoter CpG islands is associated with gene repression, DNA methylation within the gene body is associated with elevated expression [11]. This DNA methylation paradox can be partially explained by the fact that the initiation of transcription is sensitive to DNA methylation but not the transcriptional elongation [12]. Although DNA methylation is a stable epigenetic modification, it is in principle reversible and DNA methylation changes were reported in adult stem/progenitor cells such as hematopoietic stem cells or epidermal progenitor cells during differentiation and self-renewal [13,14]. With respect to HSC, it was shown that DNA methylation of specific promoters, for example phosphatase and tensin homolog (Pten) or peroxisome proliferator-activated receptor gamma (Pparγ), changed during HSC activation and fibrogenesis [15–17]. Interestingly, HSC activation can be prevented by treating isolated HSC with the demethylation agent 5'-aza-2'deoxycytidine, leading to an overall loss of methylation [17], but also by treatment with high amounts of the methyl donor SAM, an important supporting factor for DNA methylation [18]. Together these studies indicate that a balanced regulation of DNA methylation is necessary for the activation of HSC. As comprehensive analyses of DNA methylation changes are missing, our study aimed to elucidate the role of DNA methylation in quiescent and early activated HSC *in vitro*. Thereby, we wanted to gain insight into basic mechanisms of epigenetic control of quiescent adult stem cells and investigate the impact of DNA methylation changes on HSC activation, which occurs during liver regeneration and fibrogenesis.

## Materials and Methods

### Cell isolation and culture

HSC were isolated from adult male Wistar rats (>500 g), which were obtained from the animal facility of the Heinrich Heine University (Düsseldorf, Germany). The animal procedures were approved by the relevant federal authority for animal protection (Landesamt für Natur, Umwelt und Verbraucherschutz Nordrhein-Westfalen, Recklinghausen, Germany; reference number 84–02.04.2012.A344), and the animals received care according to the German animal welfare act. For HSC isolation rats were first anesthetized by ketamine/xylazine (100 mg/5 mg/kg body weight; Ketavet, PZN-3151811, Zoetis Deutschland GmbH, Berlin, Germany; Rompun, PZN-1320422, Bayer Vital GmbH, Leverkusen, Germany) and then sacrificed by exsanguination. Stellate cells were enriched by density gradient centrifugation (8% Nycodenz, Nycomed Pharma, Oslo, Norway) after enzymatic digestion of the liver essentially as described [19]. HSC were cultured in Dulbecco’s Modified Eagle Medium (DMEM, Gibco, Karlsruhe, Germany) supplemented with 10% fetal calf serum (FCS) and 1% antibiotic/antimycotic solution (Gibco). For the analysis of freshly isolated HSC (0d), cells enriched by density gradient centrifugation were also collected in Iscove’s Modified Dulbecco’s Medium (IMDM) without phenol red (Gibco) supplemented with 2% rat serum (self-made) and immediately sorted by the flow cytometer MoFlo XDP (Beckman Coulter, Krefeld, Germany). After forward and side scatter analysis, HSC were excited by UV light at 355 nm and sorted by their characteristic retinoid fluorescence at 485 nm. HSC obtained by fluorescence-activated cell sorting (FACS) were immediately used for DNA isolation without further culture. To induce a cell cycle arrest and inhibit cell replication, HSC were treated with 1 mmol/l L-mimosine (Sigma-Aldrich, St. Louis, MO, USA) in culture medium for 1–4 days. As a control treatment the same amount of phosphate buffered saline (PBS) was added to the medium.

Hepatocytes were isolated after digestion of the rat liver by collagenase CLS type II (Biochrom) perfusion and subsequent centrifugation at 60x g [20]. Hepatocytes were cultured in DMEM/F12 supplemented with 10% FCS and 1% antibiotic/antimycotic solution on collagen I coated dishes.

### Immunofluorescence staining

HSC were seeded on glass coverslips and cultured for 1–7 days in DMEM supplemented with 10% FCS. Cells were fixed with ice-cold methanol for 5 min. Blocking was performed with 10% FCS in PBS for 1h at RT and primary antibody was incubated overnight at 4°C. The primary antibody was detected with a Cy3-labeled secondary antibody (Merck Millipore, Billerica, MA, USA) and coverslips were mounted with ProLong Gold Antifade Mountant with 4,6-diamidino-2-phenylindole (DAPI, Life Technologies). IF staining was visualized with the Olympus IX50 fluorescence microscope and Cell^F software (Olympus, Tokyo, Japan). The used antibodies are specified in S1 Table.

### Global DNA methylation analysis

Genomic DNA was isolated with the DNeasy Blood & Tissue Kit (Qiagen, Hilden, Germany) according to the manufacturer's protocol. Quantification of global DNA methylation was examined by the colorimetric MethylFlash Methylated DNA Quantification Kit (Epigentek, Farmingdale, NY, USA) a methylated cytosine (5meC) ELISA (Enzyme Linked Immunosorbent Assay). The analysis was performed in triplicates with 100 ng genomic DNA per sample. Absorption at 450 nm was determined. Additional **immunofluorescence (IF) staining** of 5meC was performed in HSC cultured for 1–3 days on glass coverslips in 12-well plates and fixed with ice-cold methanol for 5 min. Cells were permeabilized for 20 min with 0.2% Triton X-100 in PBS followed by 20 min with 0.2 M HCl. Antibody incubation was performed with a monoclonal mouse antibody against 5meC (sc-56615, Santa Cruz, Dallas, TX, USA) in 1% bovine serum albumin (BSA) in PBST. For detection a Cy3-labeled secondary antibody against mouse IgG was used.

### Genome-wide DNA methylation analysis (Methyl-MiniSeq EpiQuest)

For genome-wide analysis of DNA methylation changes during HSC activation an improved version of the Reduced-Representation Bisulfite Sequencing (RRBS) using next-generation sequencing was applied [21]. This Methyl-MiniSeq EpiQuest genome-wide sequencing and data processing (ZymoResearch) was performed by BaseClear (Leiden, Netherlands). In principle, genomic DNA of freshly isolated and 3 days cultured HSC was digested by restriction endonuclease enzymes TaqαI and MspI to produce CpG-rich fragments. These fragments were ligated to adapters, recovered, subjected to bisulfite conversion and sequenced, thereby providing information about DNA methylation at a single nucleotide resolution. Furthermore, differentially methylated regions (DMRs) within CpG islands and promoter regions of genes (+/- 1kb from transcription start site) were identified. This analysis was performed with samples from one animal to provide an overview of DNA methylation changes in functionally relevant regions and to identify differentially methylated genes for subsequent methylation analysis by bisulfite sequencing in at least 3 independent HSC activation experiments.

### Gene Ontology (GO) Annotation

All genes with a DMR and a significant DNA methylation change of at least 20% were subjected to a gene ontology annotation with DAVID (Database for Annotation, Visualization and Integrated Discovery) (http://david.abcc.ncifcrf.gov) to analyze enrichment of these genes within biological processes [22]. Only GO terms with a count of at least 5 genes and p<0.01 were used.

### Bisulfite conversion and direct bisulfite sequencing

To analyze the DNA methylation of the candidate genes, DNA of freshly isolated and 3 days cultured HSC was subjected to bisulfite conversion by EpiTect Bisulfite Conversion Kit (Qiagen). Bisulfite primers were designed with MethPrimer online tool [23] covering the identified DMR (S2 Table). For amplification of bisulfite PCR products the Maxima Hot Start Master Mix (Thermo Scientific) was used with 60 ng bisulfite modified DNA and 0.6 μmol/l primers. After an initial activation step at 95°C a three step PCR protocol was used with denaturation at 95°C, annealing at 52–56°C and elongation at 72°C for 40 cycles. The PCR products were purified using Wizard SV Gel and PCR Clean Up System (Promega). Purified bisulfite PCR products were sequenced at the DNA sequencing facility of Heinrich Heine University. To quantify DNA methylation, we used the Mquant method as described [24]. In principle, the height of the thymine peak in a CpG-dinucleotide was subtracted from the average signal of 10 surrounding thymine peaks to quantify DNA methylation at this site. For our analysis we used 3–5 CpG sites and calculated the mean DNA methylation of this area.

### Quantitative PCR (qPCR)

For the analysis of gene expression we used RNA of HSC cultured for one night (quiescent) or 3 days (early activated), because reference gene expression shows a high variance in freshly isolated and cultured cells, which interferes with the normalization of qPCR data [25]. Furthermore, isolation of HSC by FACS could stress the cells and alter gene expression, therefore one night cultured HSC were used. RNA was isolated with the RNeasy Mini Kit (Qiagen) according to the manufacturer's instructions. For cDNA synthesis 400 ng total RNA was used per 20 μl reaction volume using the RevertAid H Minus First Strand cDNA Synthesis Kit (Thermo Scientific, Waltham, MA, USA). SensiMix SYBR No-ROX Kit (Bioline, Luckenwalde, Germany) was used for qPCR reaction. PCR amplification was performed with 12.5 ng cDNA and 0.6 μmol/l primers (S3 Table). After an initial denaturation at 95°C, the annealing was carried out at 56°C and elongation at 72°C for 40 cycles using TOptical cycler (Analytic Jena AG, Jena, Germany). Additional melting curve analyses were performed to validate the quality of PCR products. All samples were measured in triplicates and ribosomal protein S6 (RPS6) was used for normalization of the results obtained by the 2(-ΔΔCt) method. The expression of quiescent HSC was set to 100%.

### DNA methylation in repetitive DNA elements

To analyze the DNA methylation during early HSC activation within different repetitive elements we used the qAMP (quantitative analysis of DNA methylation using real-time PCR) method as described [26]. In principle, 300 ng genomic DNA were either restricted for 1h at 37°C with methylation-sensitive restriction enzymes like HhaI and HpaII (Thermo Scientific), which can only cleave unmethylated recognition sites, or DNA was treated without any enzyme (mock), accordingly. After digestion 2.5 ng DNA per sample were subjected to qPCR and the amount of digested DNA compared to mock-treated DNA was quantified. For primer design the consensus sequences of repetitive DNA elements were obtained from RepBase database (http://www.girinst.org/repbase) and primer3 software (http://primer3.ut.ee) was used (S4 Table). All samples were measured in triplicates and mock treated DNA was set to 100% DNA methylation.

### Western Blot analysis

For detection of proteins in HSC cultured for 0–7 days, 30–50 μg of whole protein cell lysates were separated on an 8% SDS-polyacrylamide gel and blotted. Western blots were incubated over-night with the primary antibody diluted in TBST (Tris-buffered saline with 0.1% tween 20) with 5% BSA or milk-powder. Detection was performed with a horse radish peroxidase coupled secondary antibody and WesternBright Quantum detection kit (Advansta, Menlo Park, CA, USA). A western blot with a γ-Tubulin antibody served as loading control. See S1 Table for a list of used antibodies and conditions.

### BrdU-Assay

The DNA synthesis rate of L-mimosine or control treated HSC was assessed by a BrdU-assay (5-bromo-2-deoxyuridine) after 1–4 days of culture. BrdU was added 20 h before fixation of the cells. Incorporation was determined with the Cell Proliferation ELISA BrdU Kit (Roche) according to manufacturer's protocol. Quantification of the reaction product was measured by absorption at 450nm. The measurements were performed in triplicates.

### Statistics

Significance of the data was determined by students t-test or Mann-Whitney U-test and considered significant at p<0.05. The results of at least 3 independent experiments were expressed as mean values. The variance was stated as standard error of mean (± SEM).

## Results

### Global DNA methylation during HSC activation

**Fig 1:**
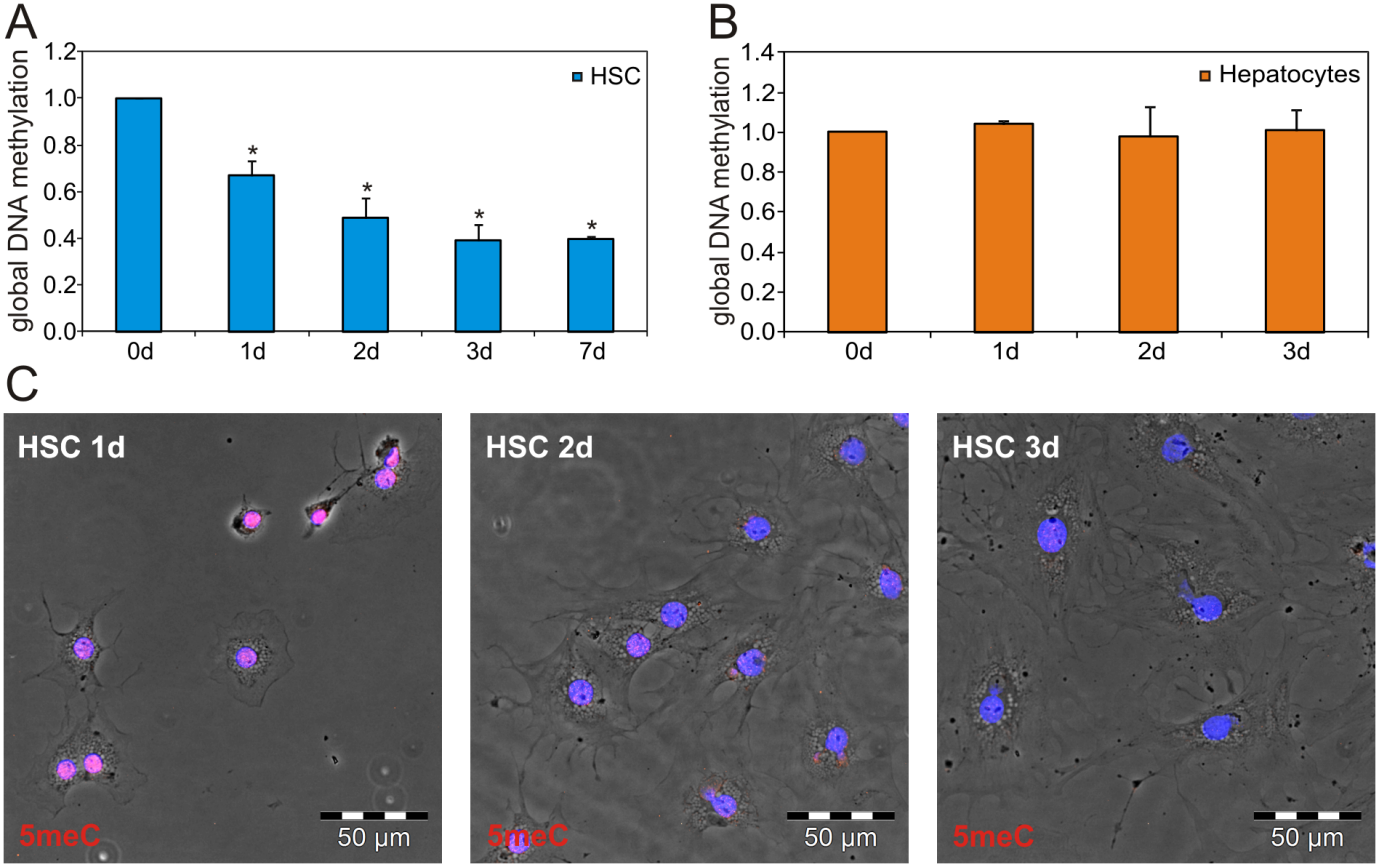
Global DNA demethylation during HSC activation. (A) Global DNA methylation determined by a 5meC ELISA showed a significant reduction to approximately 40% of the initial DNA methylation of freshly isolated HSC within 3 days of culture (* p<0.05 compared to HSC 0d, n = 4 independent experiments). (B) Global DNA methylation in hepatocytes was not altered during culture. (C) IF staining of 5meC (red) during early HSC activation. The nuclei were stained with DAPI (blue). All pictures were taken with the same exposure time and adjustments to enable direct comparison of the 5meC amount. Shown are representative images from one of 4 independent experiments.

Changes in the overall level of DNA methylation were analyzed during HSC activation in culture. Therefore, the global DNA methylation in cultured HSC was measured by a 5meC ELISA (Fig 1A). This analysis revealed a significant decrease in the 5meC amount during culture leading to a loss of approximately 60% of the initial methylation level within the first 3 days of culture. Extended culture for 7 days did not further alter the global DNA methylation of HSC, indicating that the majority of DNA demethylation was completed within 3 days of culture. In contrast to HSC, liver parenchymal cells (hepatocytes) did not show any significant changes in the global DNA methylation level during culture (Fig 1B), suggesting that the strong DNA demethylation event during HSC culture was not a general cell culture phenomenon. The loss of DNA methylation during early HSC activation was also visualized by 5meC IF staining, which showed a gradual loss of DNA methylation within the first three days of culture (Fig 1C). During this time period HSC displayed an increase in cell and nucleus size, which is characteristic for culture-activated HSC [27]. Immunofluorescence staining displayed that the activation-induced increase in αSma and Nestin protein was already visible in the early activated HSC, while quiescence-associated Gfap was decreased (S1 Fig).

### Genome-wide analysis of DNA methylation changes

**Fig 2:**
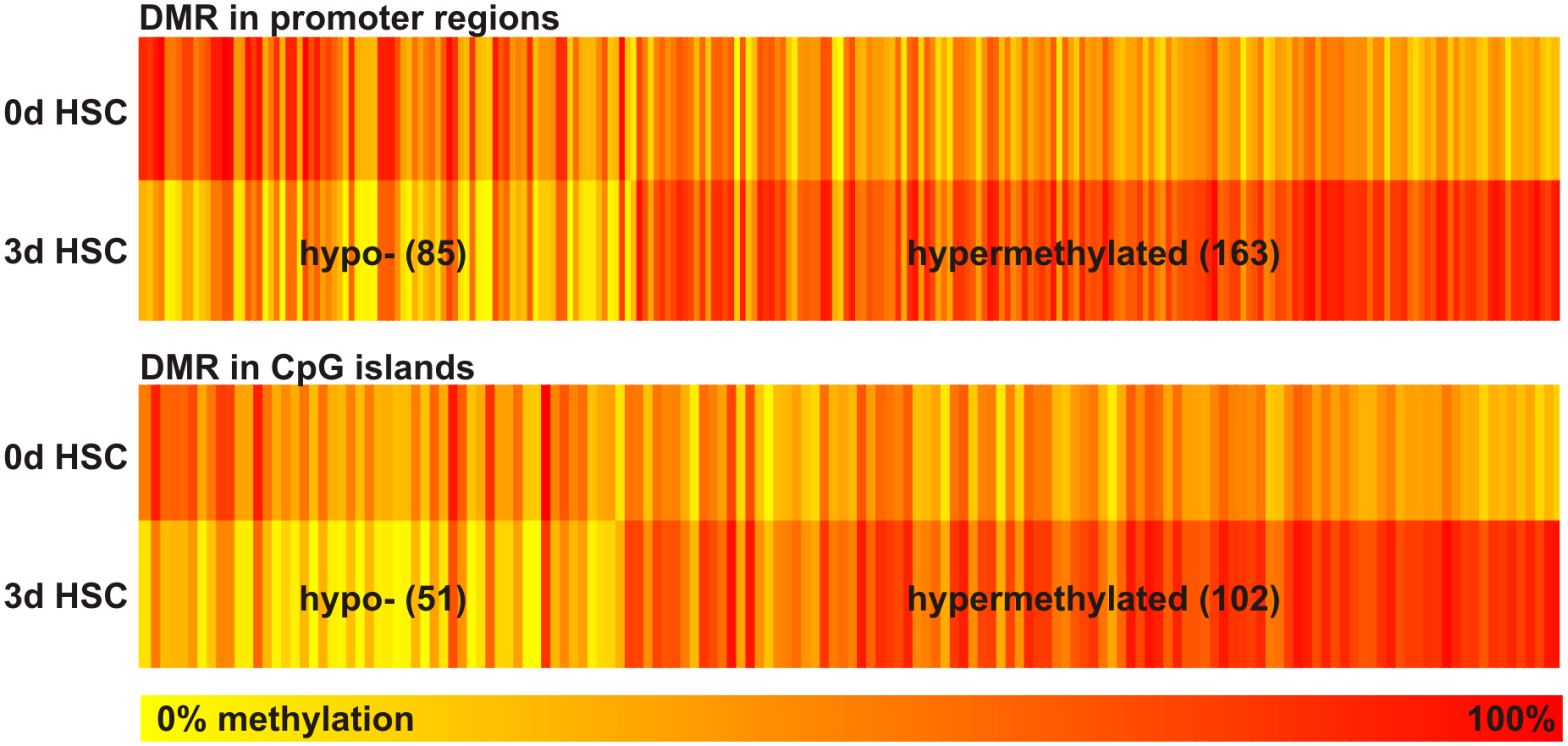
Heat map of identified differentially methylated regions (DMRs) within promoter regions or CpG islands. Only DMRs with a methylation difference of at least 20% between freshly isolated (0d) and early activated (3d) HSC and p<0.05 are displayed as a heat map. The gradual color from yellow (0%) to red (100%) indicated the level of DNA methylation according to EpiQuest sequencing. The number of DMRs was stated in brackets. There were more hypermethylated as demethylated regions identified during early HSC activation.

To find out which genes are influenced by altered DNA methylation, a genome-wide DNA methylation analysis was performed. As the global DNA methylation reached its minimum after three days of HSC culture, the genome-wide DNA methylation changes were measured in freshly isolated HSC and early activated HSC, which were maintained for 3 days in culture. Therefore, we isolated HSC by density gradient centrifugation and cultured one aliquot of cells for 3 days and the other cell aliquot was directly sorted by vitamin A dependent FACS to avoid contaminating cells in the freshly isolated fraction. Thus, values from both time points were obtained from the same animal and same preparation. The EpiQuest sequencing service from Zymo Research including full data analysis service was used to investigate genome-wide DNA methylation during early HSC activation. This method is a modified version of the RRBS protocol and enables the analysis of CpG-rich regions. It provides a coverage of up to 90% of functionally relevant annotated regions such as CpG islands and gene promoters, which are of special importance with respect to epigenetic gene regulation, while genomic coverage is only around 10–12% [28]. CpG methylation was measured throughout the genome in all chromosomes, while non-CpG methylation was not found (S2 Fig). This indicated that bisulfite conversion and RRBS did work properly. The whole sequencing results were visualized with the UCSC genome browser (https://genome.ucsc.edu) and can be observed using the supplied links of genome browser tracks in S5 Table. To identify genes which were regulated by DNA methylation changes, a DMR (differentially methylated region) analysis of promoter regions and CpG islands was performed. Only significant DMRs with a methylation change of at least 20% were used for further analysis, which led to the identification of 248 DMRs in promoter regions and 153 in CpG islands (S6 Table). The heat maps of the DMR analysis revealed that there was DNA hypo- as well as hypermethylation during early HSC activation (Fig 2). Interestingly, EpiQuest sequencing identified twice as much DMRs with DNA hypermethylation as with hypomethylation, which is contrary to the measured global DNA methylation changes. To investigate if the genes associated with DMR may be important for HSC activation, a gene ontology (GO) annotation with DAVID was performed. 298 out of 329 genes were identified by DAVID and were used to find enriched GO terms with respect to biological processes. The 20 entries of enriched GO terms with the highest significance level included "regulation of cell activation", "immune response", "response to wounding" and "regulation of localization", which are relevant processes during HSC activation (Table 1, S7 Table).

**Table 1:** List of the first 20 GO terms obtained by DAVID from identified differentially methylated genes altered during early *in vitro* activation of HSC sorted by p value.

GO ID	GO Term	Count	Fold Enrich.	PValue
GO:0051239	regulation of multicellular organismal process	40	2,44	2,54E-07
GO:0065007	biological regulation	125	1,39	8,45E-07
GO:0050789	regulation of biological process	118	1,42	1,16E-06
GO:0002694	regulation of leukocyte activation	14	5,25	2,58E-06
GO:0050865	regulation of cell activation	14	5,03	4,15E-06
GO:0051249	regulation of lymphocyte activation	13	5,45	4,56E-06
GO:0002682	regulation of immune system process	20	3,20	1,54E-05
GO:0006955	immune response	21	3,06	1,69E-05
GO:0050794	regulation of cellular process	108	1,38	2,20E-05
GO:0007165	signal transduction	49	1,83	2,97E-05
GO:0006954	inflammatory response	14	3,89	6,36E-05
GO:0002683	negative regulation of immune system process	9	6,46	7,15E-05
GO:0006952	defense response	19	2,87	1,09E-04
GO:0009605	response to external stimulus	29	2,15	1,74E-04
GO:0002695	negative regulation of leukocyte activation	7	7,97	2,26E-04
GO:0009611	response to wounding	19	2,70	2,32E-04
GO:0003013	circulatory system process	11	4,28	2,47E-04
GO:0008015	blood circulation	11	4,28	2,47E-04
GO:0044057	regulation of system process	16	3,03	2,65E-04
GO:0032879	regulation of localization	24	2,31	2,68E-04

### Analysis of newly identified differentially methylated genes

**Fig 3:**
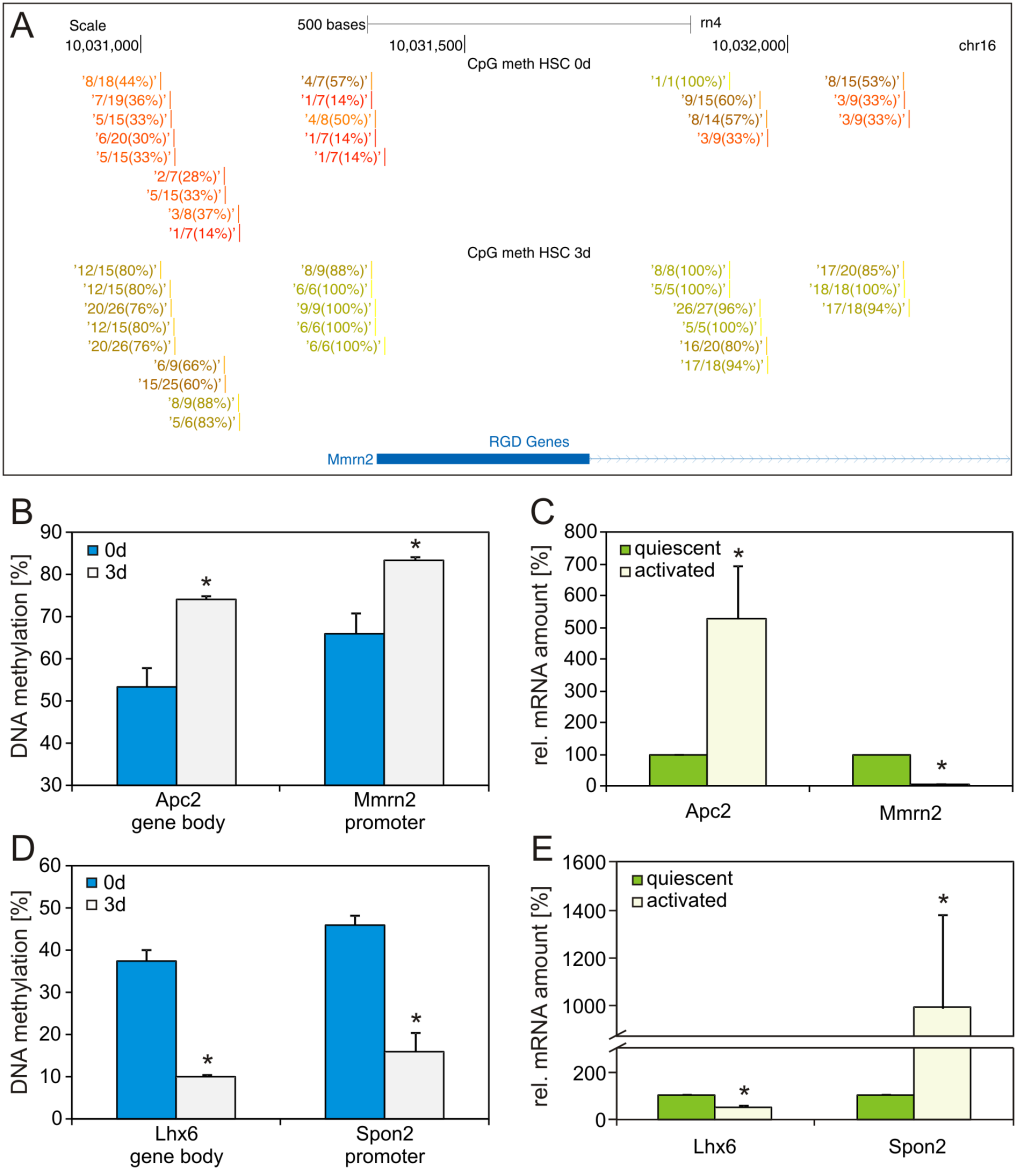
DNA methylation and expression analysis of genes identified by EpiQuest sequencing. (A) EpiQuest sequencing result of Mmrn2 promoter region displayed in UCSC genome browser. DNA methylation at individual CpG dinucleotides within the sequence are specified in percent. (B+D) DNA methylation analysis of two hyper- and hypomethylated genes by direct bisulfite sequencing (n = 3 independent experiments). The position of the analyzed region is indicated in the figure (* p<0.05). (C+E) Corresponding quantitative gene expression analysis to determine the effect of DNA methylation changes on the gene expression in quiescent (cultured overnight) and early activated (cultured for 3 days) HSC (n = 5 independent experiments).

DNA methylation changes found by EpiQuest sequencing were visualized with the UCSC genome browser (Fig 3a) displaying the mean DNA methylation and the read coverage at each analyzed CpG dinucleotide. To validate these EpiQuest results DNA methylation changes of selected genes identified by DMR analysis were investigated by direct bisulfite sequencing of freshly isolated and 3 days cultured HSC. To correlate DNA methylation changes with changes in gene expression, qPCR analysis was performed. The genes adenomatosis polyposis coli 2 (Apc2) and multimerin 2 (Mmrn2) were methylated according to EpiQuest sequencing. Direct bisulfite sequencing confirmed an increase in DNA methylation of approximately 20% in both genes (Fig 3B). Additional gene expression analysis revealed a significant increase in Apc2 expression and a decrease in Mmrn2 (Fig 3C). This correlated well with the DNA methylation changes as Apc2 was methylated within the gene body, which is associated with increased expression, while Mmrn2 showed a promoter methylation, which can reduce expression. Next we analyzed genes which were hypomethylated during early HSC activation. The EpiQuest results of LIM homeobox protein 6 (Lhx6) gene body and Spondin2 (Spon2) promoter methylation could also be confirmed by direct bisulfite sequencing and showed a decrease in DNA methylation of approximately 30% (Fig 3D). The gene expression analysis demonstrated a decrease in Lhx6 and an increase in Spon2 expression during early HSC activation (Fig 3E), which again showed a good correlation between DNA methylation and expression. DNA methylation and gene expression were measured for 10 genes identified by EpiQuest sequencing. Changes in DNA methylation due to HSC activation could be confirmed in all 10 genes and correlated with gene expression changes, whereas DNA methylation at promoter regions and 3'UTR (three prime untranslated region) led to decreased gene expression, while DNA methylation at the gene body supported expression (Table 2, S3 Fig).

**Table 2:** Overview of the DNA methylation and gene expression results of 10 analyzed genes identified by EpiQuest sequencing.

gene	meth. diff. EpiQuest	meth. diff. bisulfite seq.	position	expression
Apc2	34%	21%	gene body	increased
Cnr2	63%	38%	promoter	decreased
Inpp5d	38%	24%	promoter	decreased
Klf2	52%	34%	3' UTR	decreased
Lhx6	-24%	-28%	gene body	decreased
miR126	36%	24%	promoter	decreased
Mmrn2	50%	18%	promoter	decreased
Robo4	41%	33%	promoter	decreased
Spon2	-31%	-30%	promoter	increased
Wnt5a	26%*	20%	gene body	increased

Detailed bisulfite sequencing and expression results of all analyzed genes are shown in S3 Fig.

* Wnt5a was identified by manual evaluation of EpiQuest sequencing data.

### DNA methylation at repetitive DNA elements

**Fig 4:**
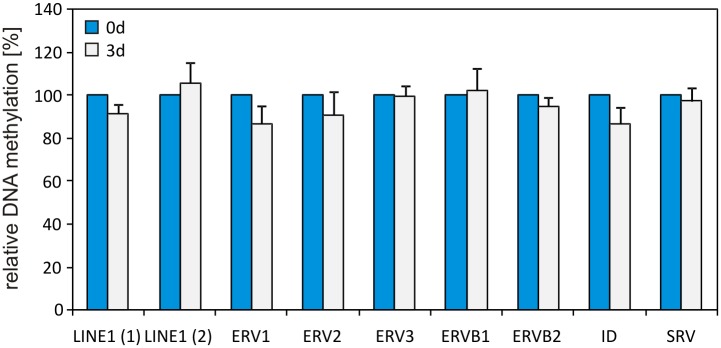
DNA methylation analysis in repetitive DNA elements. The DNA methylation was measured within consensus sequences of different repetitive elements by qAMP (n = 5). DNA methylation of freshly isolated HSC (0d) was set to 100%. There were no significant changes in the DNA methylation level between freshly isolated and 3d cultured HSC detectable.

The detected difference between global DNA demethylation and increased DNA methylation in CpG-rich regions measured by EpiQuest sequencing, could be explained by DNA demethylation in regions of the genome, which were not covered by the EpiQuest sequencing. One possibility is that the DNA demethylation could take place at repetitive DNA elements, which account for approximately 40% of the rat genome [29]. Therefore, changes in DNA methylation level at different repetitive DNA elements during early HSC activation were determined by qAMP. For the analysis consensus sequences of different elements provided by RepBase were used, this included LINE (long interspersed nuclear element), SINE (short interspersed nuclear element) like ID (identifier) elements and SRV (simian retrovirus) as well as different LTR (long terminal repeat) elements from ERVs (endogenous retroviruses). The analysis showed no significant changes in the DNA methylation of these repetitive DNA elements (Fig 4). Thus, the majority of DNA demethylation during early HSC activation may have occurred in CpG-poor regions, which were neither covered by EpiQuest sequencing nor by qAMP of repetitive DNA elements.

### Active DNA demethylation during early HSC activation

**Fig 5:**
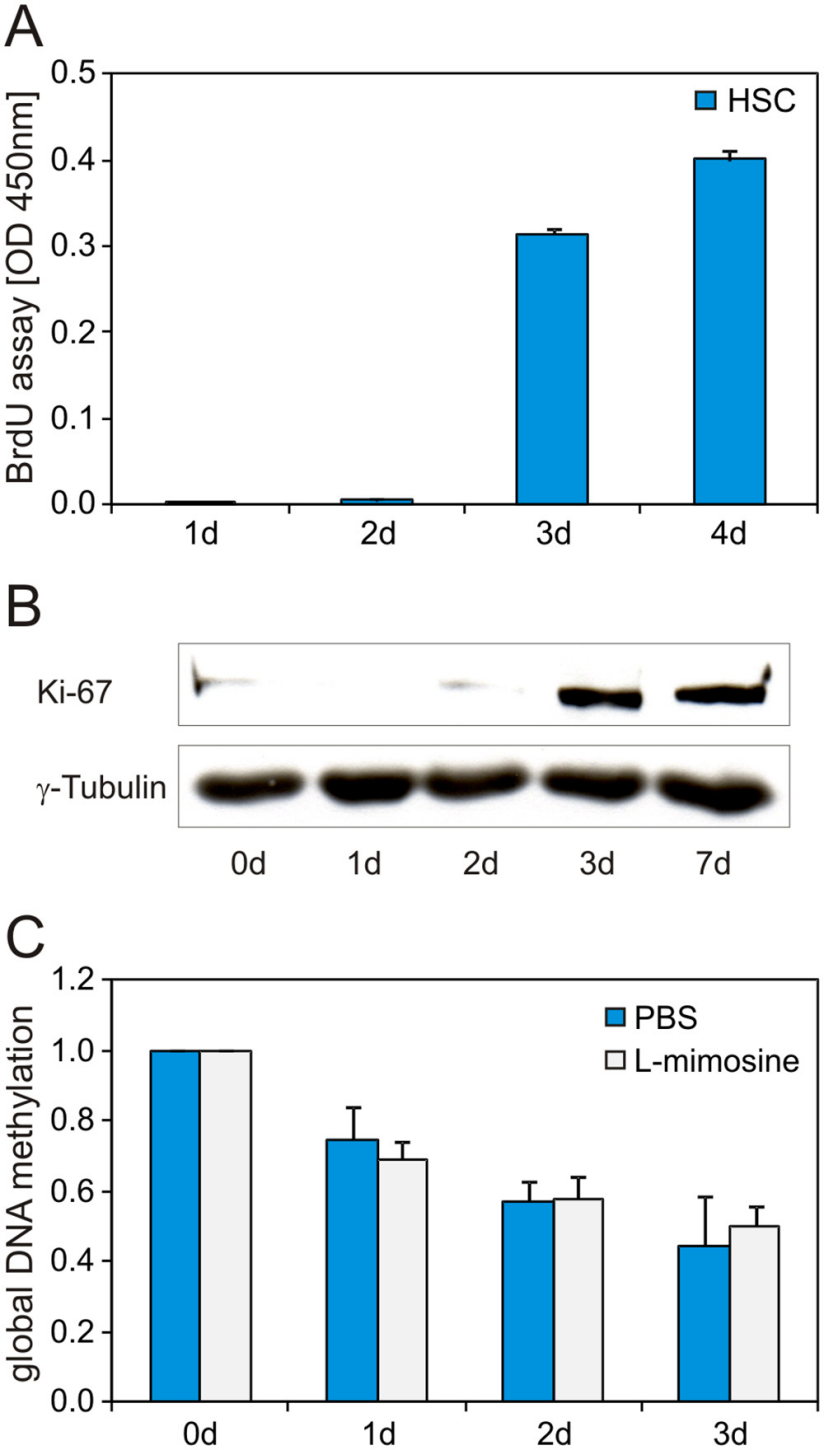
Global DNA demethylation during early HSC activation is due to an active mechanism. (A) BrdU assay during HSC culture showed that DNA synthesis in HSC was absent during the first two days of culture. (B) In line with this, the Ki67 western blot revealed that freshly isolated HSC were in the G0-phase and entered the cell cycle at the third day of culture. (C) L-mimosine treated HSC showed global DNA demethylation during culture with no significant difference to control treated cells (n = 3 independent experiments). Thus DNA demethylation was independent of DNA synthesis implicating an active mechanism. Global DNA methylation was determined by a 5meC ELISA.

It was further investigated if DNA demethylation during early HSC activation depended on an enzyme-based active or a passive demethylation mechanism, which is facilitated through repetitive cell divisions without methylating the newly synthesized DNA strand. As passive demethylation is based on DNA synthesis, the DNA synthesis was analyzed in HSC during culture by BrdU incorporation. The BrdU assay revealed that DNA synthesis started in HSC at the third day of culture. No significant DNA synthesis was detectable during the first two days of culture (Fig 5A). This result was supported by western blot analysis of Ki67. Freshly isolated HSC had only low Ki67 protein amounts, but Ki67 increased after three days of culture (Fig 5B). Thus, HSC were predominantly in G0-phase after isolation and entered the cell cycle after 3 days of culture. To further exclude passive DNA demethylation mechanism, we treated HSC with L-mimosine which induced a cell-cycle arrest and inhibited DNA synthesis (S4 Fig) and measured the global DNA methylation during early HSC activation. L-mimosine treated HSC showed a global DNA demethylation during HSC culture which was comparable to PBS control treated cells (Fig 5C). As DNA methylation decreased rapidly within the first two days of culture and no DNA synthesis was detectable or even excluded experimentally by L-mimosine treatment, an active DNA demethylation mechanism seemed to be responsible for global DNA demethylation.

### Analysis of DNA methyltransferases (Dnmt) during HSC activation

**Fig 6:**
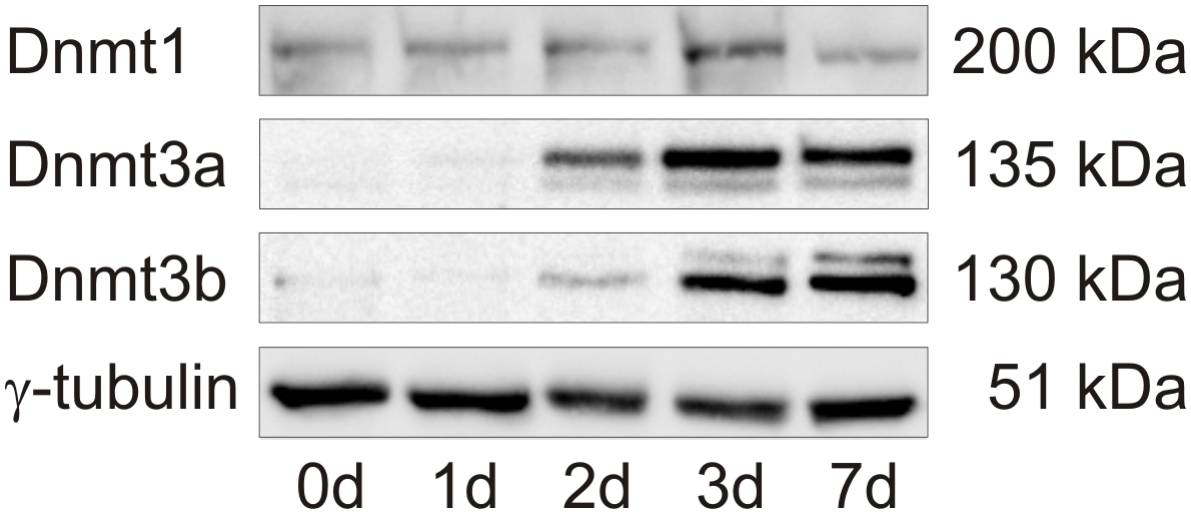
Western blot analysis of Dnmts during HSC activation. Exemplary results during a HSC time course experiment. Protein levels were determined in freshly isolated and 1, 2, 3 or 7 days cultured HSC. The analysis showed an overall increase of the *de novo* DNA methyltransferases Dnmt3a and Dnmt3b. Detection of γ-tubulin served as loading control.

As Dnmts are essential proteins for the integrity of DNA methylation during replication but also for the induction of new DNA methylation, we analyzed the protein level of the maintenance DNA methytransferase Dnmt1 and *de novo* methyltransferases Dnmt3a and Dnmt3b. This analysis showed a strong increase in Dnmt3a and Dnmt3b during activation, while these enzymes were almost not detectable in freshly isolated HSC on day 0 (Fig 6). Highest protein levels of Dnmt3a and Dnmt3b were found on day 3 and day 7, respectively, and could be responsible for the gene-specific DNA hypermethylation. The maintenance DNA methyltransferase Dnmt1 was detectable throughout HSC activation but was increased on the third day of culture (Fig 6). During this time DNA synthesis is started, which may account for this higher Dnmt1 level.

## Discussion

Although first studies exist that HSC activation is regulated by epigenetic mechanisms [30–32] and even new therapeutic options for treating liver diseases have been proposed based on these epigenetic mechanisms [33,34], the knowledge about DNA methylation changes during HSC activation is still limited. It has been reported that the DNA methylation inhibitor 5-aza-2-deoxycytidine can block HSC activation *in vitro*, which was assigned to the inhibition of DNA hypermethylation at specific genes such as Pten, Smad7 or IkBa (NF-kappa-B inhibitor alpha) [15,17,35], while other genes associated with HSC activation such as Nestin remained unmethylated and were controlled by histone modifications [16]. However, the whole extent of DNA methylation changes during HSC activation remained unknown. This study reveals that the first days of culture-induced HSC activation are accompanied by strong changes in the DNA methylation profile. Although HSC activation led to an overall loss of DNA methylation, we could demonstrate gene-specific DNA hyper- and hypomethylation in CpG-rich regions inducing altered gene expression in early activated cells. GO annotation of differentially methylated genes showed enrichment in GO terms, which were associated with biological processes like wound healing, inflammation, migration and cell activation, which are important events during HSC activation [36]. This implicates that the detected DNA methylation changes control essential mechanisms of HSC activation. This finding was also supported by the analysis of the identified candidate genes Apc2 and Wnt5a. Both genes are players in different Wnt signaling pathways and during *in vitro* activation of HSC as well as during liver fibrosis an increased Wnt5a expression has been reported [37,38]. This is in line with our results which revealed an up-regulation of both genes by DNA methylation changes. Furthermore, it has been reported that HSC activation is accompanied by a switch from canonical to non-canonical Wnt pathways [39]. The increased expression of Apc2, a known negative regulator of canonical Wnt pathway, and Wnt5a, a ligand and mediator of non-canonical Wnt pathways, supports this concept. Other epigenetic mechanisms can also be influenced by DNA methylation changes, for example the pri-miRNA form of miRNA126 was down-regulated during early HSC activation, which was associated with increased DNA methylation at its promoter. It is known that miRNA126 is a negative regulator of vascular endothelial growth factor (Vegf) signal transduction and decreases proliferation in HSC [40]. Thus miRNA126 down-regulation supports HSC activation. Another hallmark of HSC activation is the increased expression of extracellular matrix proteins. In our study early HSC activation led to increased expression of extracellular matrix protein Spon2, which was regulated by DNA hypomethylation of Spon2 promoter. Thus we were able to identify several gene-specific DNA hyper- as well as hypomethylations which may control essential processes and signaling pathways during HSC activation. This could also explain why inhibition of methylation by 5'-aza-2'-deoxycytidin impedes HSC activation. In contrast to this, we could not detect significant methylation changes in repetitive elements. Especially LINE-1 methylation which is often used to determine global DNA methylation changes is not altered despite the overall loss of DNA methylation. This is in line with an analysis in patients with cirrhosis, which also showed no differences in LINE-1 methylation between normal and diseased livers, despite high numbers of activated stellate cells in cirrhotic livers [41]. Furthermore, a recently published meta-analysis of different cancer types could not detect altered LINE-1 methylation in hepatocellular carcinoma patients [42]. Thus *in vivo* activation of HSC does not alter LINE-1 DNA methylation analog to our *in vitro* experiments.

Interestingly, genome-wide EpiQuest sequencing showed more DNA hyper- than hypomethylated regions during early HSC activation while global DNA methylation decreases. As the DNA methylation in the analyzed repetitive DNA elements was not altered and EpiQuest sequencing was restricted to CpG-rich regions, the observed global DNA demethylation may have occurred at CpG-poor regions. DNA methylation changes in CpG-poor regions are also able to influence gene expression [43], thus additional studies are required to investigate these regions. Nonetheless, global loss of DNA methylation is generally linked to increased differentiation potential in stem cells and was described during embryonic development [44]. Furthermore, DNA demethylation by 5'-aza-2'-deoxycytidin treatment can be used during the generation of induced pluripotent stem cells to increase their frequency [45]. Thus global DNA demethylation is probably necessary to enable cell differentiation processes in HSC. This is supported by the finding that treatment with the methyl donor SAM, which counteracts DNA demethylation, was reported to inhibit HSC activation [18,46]. On the other hand gene-specific hypermethylation is also important during activation and could be attributed to the increase of *de novo* DNA methyltransferases Dnmt3a and Dnmt3b. The latter has been reported to be responsible for the Pten hypermethylation during HSC activation [47]. Taken together this study revealed that culture-dependent HSC activation is regulated by coordinated DNA methylation and DNA demethylation to establish an activation-specific gene expression pattern. This may also be an important event during *in vivo* HSC activation in liver regeneration and fibrosis, which needs further investigation.

Finally, our data suggest that the global loss of DNA methylation is based on an active DNA demethylation mechanism, whereby demethylation is tightly controlled. Repetitive DNA elements for instance were protected against DNA demethylation during early activation presumably to maintain genomic stability and prevent retrotransposition of these elements [48]. Active DNA demethylation has been identified during embryonic development and is thought to play a role during stem cell differentiation, but appropriate models to analyze active DNA demethylation are limited [49,50]. Here we show that *in vitro* activation of HSC is accompanied by a strong loss of DNA methylation by an active DNA demethylation mechanism, making it a versatile model to analyze this process in adult stem cells, which is still not completely elucidated [51].

In summary, we were able to show that early activation of isolated HSC is controlled by coordinated, active DNA methylation and demethylation events which regulate the gene expression of factors associated with developmental as well as fibrotic processes. These findings may lead to a better knowledge of HSC biology and may offer novel approaches to understand impaired liver regeneration and the development of liver fibrosis. Furthermore, *in vitro* activation of HSC can serve as a suitable model to analyze the active DNA demethylation mechanism and can provide new insight into the control of quiescence and activation in adult stem cells.

## Supporting Information

S1 Fig Immunofluorescence staining during *in vitro* activation of HSC. Vitamin A content of HSC decreased with culture time as displayed by the autofluorescence of vitamin A (blue). IF staining of αSma, Gfap, Nestin and Desmin (red) showed an increase in activation-associated factors αSma and Nestin during culture, while Gfap decreased. The nuclei were stained with DAPI (blue).
(TIF)

S2 Fig Overview and quality control of EpiQuest sequencing results. (A+B) Overview of DNA methylation measured within CpG-dinucleotides in HSC 0d and 3d throughout the genome. No significant amount of non-CpG methylation were detected throughout the genome, which indicates that the bisulfite modification and subsequent EpiQuest sequencing worked properly. (C+D) These diagrams display the coverage of the analyzed sequences at the different chromosomes for both samples showing that all chromosome were at least partially included in the analysis. (E) Table of the quality statistics of the EpiQuest sequencing.
(TIF)

S3 Fig DNA methylation and gene expression analysis of 10 genes identified by EpiQuest sequencing. (A) DNA methylation was determined by direct bisulfite sequencing and showed significant changes of DNA methylation during *in vitro* activation (n = 3 independent experiments; * p<0.05). (B) The corresponding quantitative gene expression analysis was determined with quiescent (cultured overnight) and early activated (3 days cultured) HSC (n = 5 independent experiments; * p<0.05). These analyses revealed a good correlation between DNA methylation changes and gene expression, if the position of the DNA methylation was included (Table 2).
(TIF)

S4 Fig L-mimosine treatment led to an inhibition of DNA synthesis in HSC. The DNA synthesis of L-mimosine or PBS control treated HSC in culture were measured with a BrdU assay (n = 3). The analysis revealed that the L-mimosine treatment almost completely blocked the DNA synthesis in cultured HSC while DNA synthesis was induced at day 3 of culture in control treated cells.
(TIF)

S1 Table List of antibodies and used conditions for immunofluorescence staining and western blot. (PDF)

S2 Table List of bisulfite sequencing primers. (PDF)

S3 Table List of gene expression primers. (PDF)

S4 Table List of primers used for qAMP of repetitive DNA elements. (PDF)

S5 Table UCSC genome browser tracks obtained from EpiQuest sequencing for rat genome browser assembly Baylor3.4/rn4. (PDF)

S6 Table Excel file containing all DMRs identified by EpiQuest sequencing in CpG-islands and promoter regions during HSC activation with a methylation difference of at least 20% and p<0.05. (XLSX)

S7 Table Complete list of GO terms generated by DAVID with differentially methylated genes during HSC activation sorted by p-value (count >5, p<0.01). (PDF)

## References

[1] Kordes C, Sawitza I, Götze S, Häussinger D. Hepatic stellate cells support hematopoiesis and are liver-resident mesenchymal stem cells. Cell Physiol Biochem. 2013;31:290–304. 10.1159/000343368 23485574

[2] Kordes C, Sawitza I, Götze S, Herebian D, Häussinger D. Hepatic stellate cells contribute to progenitor cells and liver regeneration. J Clin Invest. 2014;124:5503–15. 10.1172/JCI74119 25401473 PMC4348953

[3] Friedman SL, Roll FJ, Boyles J, Bissell DM. Hepatic lipocytes: the principal collagen-producing cells of normal rat liver. Proc. Natl. Acad. Sci. U.S.A. 1985;82:8681–8685. 3909149 10.1073/pnas.82.24.8681 PMC391500

[4] Sawitza I, Kordes C, Reister S, Häussinger D. The niche of stellate cells within rat liver. Hepatology. 2009;50:1617–1624. 10.1002/hep.23184 19725107

[5] Kordes C, Häussinger D. Hepatic stem cell niches. J Clin Invest. 2013;123:1874–80 10.1172/JCI66027 23635785 PMC3638908

[6] Gard A, White F, Dutton G. Extra-neural glial fibrillary acidic protein (GFAP) immunoreactivity in perisinusoidal stellate cells of rat liver. J. Neuroimmunol. 1985;8:359–375. 3891783 10.1016/s0165-5728(85)80073-4

[7] Yokoi Y, Namihisa T, Kuroda H, Komatsu I, Miyazaki A, Watanabe S, et al Immunocytochemical detection of desmin in fat-storing cells (Ito cells). Hepatology. 1984;4:709–714. 6204917 10.1002/hep.1840040425

[8] Castilho-Fernandes A, de Almeida DC, Fontes AM, Melo FU, Picanço-Castro V, Freitas MC, et al Human hepatic stellate cell line (LX-2) exhibits characteristics of bone marrow-derived mesenchymal stem cells. Exp Mol Pathol. 2011;91:664–672. 10.1016/j.yexmp.2011.09.002 21930125

[9] Ishikawa T, Factor VM, Marquardt JU, Raggi C, Seo D, Kitade M, et al Hepatocyte growth factor/c-met signaling is required for stem-cell-mediated liver regeneration in mice. Hepatology. 2012;55:1215–26. 10.1002/hep.24796 22095660 PMC3299882

[10] Chuang JC, Jones PA. Epigenetics and microRNAs. Pediatr Res. 2007;61:24R–29R 17413852 10.1203/pdr.0b013e3180457684

[11] Jones PA. Functions of DNA methylation: islands, start sites, gene bodies and beyond. Nat Rev Genet. 2012;13:484–92. 10.1038/nrg3230 22641018

[12] Jones PA. The DNA methylation paradox. Trends Genet. 1999;15:34–7. 10087932 10.1016/s0168-9525(98)01636-9

[13] Bröske A, Vockentanz L, Kharazi S, Huska MR, Mancini E, Scheller M, et al DNA methylation protects hematopoietic stem cell multipotency from myeloerythroid restriction. Nat Genet. 2009;41:1207–1215. 10.1038/ng.463 19801979

[14] Sen GL, Reuter JA, Webster DE, Zhu L, Khavari PA. DNMT1 maintains progenitor function in self-renewing somatic tissue. Nature. 2010;463:563–567. 10.1038/nature08683 20081831 PMC3050546

[15] Bian EB, Huang C, Ma TT, Tao H, Zhang H, Cheng C, et al DNMT1-mediated PTEN hypermethylation confers hepatic stellate cell activation and liver fibrogenesis in rats. Toxicol Appl Pharmacol. 2012;264:13–22. 10.1016/j.taap.2012.06.022 22841775

[16] Reister S, Kordes C, Sawitza I, Häussinger D. The epigenetic regulation of stem cell factors in hepatic stellate cells. Stem Cells Dev. 2011;20:1687–99. 10.1089/scd.2010.0418 21219128

[17] Mann J, Oakley F, Akiboye F, Elsharkawy A, Thorne AW, Mann DA. Regulation of myofibroblast transdifferentiation by DNA methylation and MeCP2: implications for wound healing and fibrogenesis. Cell Death Differ. 2007;14:275–85. 16763620 10.1038/sj.cdd.4401979

[18] Matsui H, Kawada N. Effect of S-adenosyl-L-methionine on the activation, proliferation and contraction of hepatic stellate cells. Eur J Pharmacol. 2005;509:31–36. 15713426 10.1016/j.ejphar.2004.12.041

[19] Hendriks HF, Verhoofstad WA, Brouwer A, de Leeuw AM, Knook DL. Perisinusoidal fat-storing cells are the main vitamin A storage sites in rat liver. Exp Cell Res. 1985;160:138–49. 4043241 10.1016/0014-4827(85)90243-5

[20] Berry MN, Friend DS. High-yield preparation of isolated rat liver parenchymal cells: a biochemical and fine structural study. J Cell Biol. 1969;43:506–20. 4900611 10.1083/jcb.43.3.506 PMC2107801

[21] Meissner A, Gnirke A, Bell GW, Ramsahoye B, Lander ES, Jaenisch R. Reduced representation bisulfite sequencing for comparative high-resolution DNA methylation analysis. Nucleic Acids Res. 2005;33:5868–77. 16224102 10.1093/nar/gki901 PMC1258174

[22] Huang DW, Sherman BT, Lempicki RA. Systematic and integrative analysis of large gene lists using DAVID bioinformatics resources. Nature Protocols. 2009;4:44–57. 10.1038/nprot.2008.211 19131956

[23] Li LC, Dahiya R. MethPrimer: designing primers for methylation PCRs. Bioinformatics. 2002;18:1427–31. 12424112 10.1093/bioinformatics/18.11.1427

[24] Leakey TI, Zielinski J, Siegfried RN, Siegel ER, Fan CY, Cooney CA. A simple algorithm for quantifying DNA methylation levels on multiple independent CpG sites in bisulfite genomic sequencing electropherograms. Nucleic Acids Res. 2008;36:e64 10.1093/nar/gkn210 18480118 PMC2441810

[25] Lakner AM, Moore CC, Gulledge AA, Schrum LW. Daily genetic profiling indicates JAK/STAT signaling promotes early hepatic stellate cell transdifferentiation. World J Gastroenterol. 2010;16:5047–5056. 20976841 10.3748/wjg.v16.i40.5047 PMC2965281

[26] Oakes CC, La Salle S, Robaire B, Trasler JM. Evaluation of a quantitative DNA methylation analysis technique using methylation-sensitive/dependent restriction enzymes and real-time PCR. Epigenetics. 2006;1:146–52. 17965615 10.4161/epi.1.3.3392

[27] Friedman SL, Roll FJ, Boyles J, Arenson DM, Bissell DM. Maintenance of differentiated phenotype of cultured rat hepatic lipocytes by basement membrane matrix. J Biol Chem. 1989;264:10756–62. 2732246

[28] Bock C, Tomazou EM, Brinkman AB, Müller F, Simmer F, Gu H, et al Quantitative comparison of genome-wide DNA methylation mapping technologies. Nat Biotechnol. 2010;28:1106–14. 10.1038/nbt.1681 20852634 PMC3066564

[29] Gibbs RA, Weinstock GM, Metzker ML, Muzny DM, Sodergren EJ, Scherer S, et al Genome sequence of the Brown Norway rat yields insights into mammalian evolution. Nature. 2004;428:493–521. 15057822 10.1038/nature02426

[30] Mann DA, Mann J. Epigenetic regulation of hepatic stellate cell activation. J Gastroenterol Hepatol. 2008;23: Suppl 1:S108–11. 10.1111/j.1440-1746.2007.05295.x 18336652

[31] Tsukamoto H, Zhu NL, Asahina K, Mann DA, Mann J. Epigenetic cell fate regulation of hepatic stellate cells. Hepatol Res. 2011;41:675–82. 10.1111/j.1872-034X.2011.00804.x 21504520 PMC4321904

[32] Zhao Q, Qin CY, Zhao ZH, Fan YC, Wang K. Epigenetic modifications in hepatic stellate cells contribute to liver fibrosis.Tohoku J Exp Med. 2013;229:35–43. 23238615 10.1620/tjem.229.35

[33] Zeybel M, Mann DA, Mann J. Epigenetic modifications as new targets for liver disease therapies. J Hepatol. 2013;59:1349–53. 10.1016/j.jhep.2013.05.039 23747756

[34] Bian EB, Huang C, Wang H, Wu BM, Zhang L, Lv XW, et al DNA methylation: new therapeutic implications for hepatic fibrosis. Cell Signal. 2013;25:355–8. 10.1016/j.cellsig.2012.10.007 23085259

[35] Bian EB, Huang C, Wang H, Chen XX, Zhang L, Lv XW, et al Repression of Smad7 mediated by DNMT1 determines hepatic stellate cell activation and liver fibrosis in rats. Toxicol Lett.http://www.ncbi.nlm.nih.gov/pubmed/?term=repression+of+Smad7+hepatic+stellate+cell 2014;224:175–85. 10.1016/j.toxlet.2013.10.038 24211420

[36] Pellicoro A, Ramachandran P, Iredale JP, Fallowfield JA. Liver fibrosis and repair: immune regulation of wound healing in a solid organ. Nat Rev Immunol. 2014;14:181–94. 10.1038/nri3623 24566915

[37] Jiang F, Parsons CJ, Stefanovic B. Gene expression profile of quiescent and activated rat hepatic stellate cells implicates Wnt signaling pathway in activation. J Hepatol. 2006;45:401–9. 16780995 10.1016/j.jhep.2006.03.016

[38] Rashid ST, Humphries JD, Byron A, Dhar A, Askari JA, Selley JN, et al Proteomic analysis of extracellular matrix from the hepatic stellate cell line LX-2 identifies CYR61 and Wnt-5a as novel constituents of fibrotic liver. J Proteome Res. 2012;11:4052–64. 10.1021/pr3000927 22694338 PMC3411196

[39] Kordes C, Sawitza I, Häussinger D. Canonical Wnt signaling maintains the quiescent stage of hepatic stellate cells. Biochem Biophys Res Commun. 2008;367:116–23. 18158920 10.1016/j.bbrc.2007.12.085

[40] Guo CJ, Pan Q, Xiong H, Qiao YQ, Bian ZL, Zhong W, et al Dynamic expression of miR-126* and its effects on proliferation and contraction of hepatic stellate cells. FEBS Lett. 2013;587:3792–801. 10.1016/j.febslet.2013.09.047 24140635

[41] Gao XD, Qu JH, Chang XJ, Lu YY, Bai WL, Wang H, et al Hypomethylation of long interspersed nuclear element-1 promoter is associated with poor outcomes for curative resected hepatocellular carcinoma. Liver Int. 2014;34:136–46. 23875825 10.1111/liv.12264 PMC4238827

[42] Barchitta M, Quattrocchi A, Maugeri A, Vinciguerra M, Agodi A. LINE-1 hypomethylation in blood and tissue samples as an epigenetic marker for cancer risk: a systematic review and meta-analysis. PLoS One. 2014;9:e109478 10.1371/journal.pone.0109478 25275447 PMC4183594

[43] Nagae G, Isagawa T, Shiraki N, Fujita T, Yamamoto S, Tsutsumi S, et al Tissue-specific demethylation in CpG-poor promoters during cellular differentiation. Hum Mol Genet. 2011;20:2710–21. 10.1093/hmg/ddr170 21505077

[44] Hajkova P, Erhardt S, Lane N, Haaf T, El-Maarri O, Reik W, et al Epigenetic reprogramming in mouse primordial germ cells. Mech Dev. 2002;117:15–23. 12204247 10.1016/s0925-4773(02)00181-8

[45] Feng B, Ng J, Heng JD, Ng H. Molecules that promote or enhance reprogramming of somatic cells to induced pluripotent stem cells. Cell Stem Cell. 2009;4:301–312. 10.1016/j.stem.2009.03.005 19341620

[46] Zhang F, Zhuge YZ, Li YJ, Gu JX. S-adenosylmethionine inhibits the activated phenotype of human hepatic stellate cells via Rac1 and matrix metalloproteinases. Int Immunopharmacol. 2014;19:193–200. 10.1016/j.intimp.2014.01.021 24495518

[47] Zheng J, Wu C, Lin Z, Guo Y, Shi L, Dong P, et al Curcumin up-regulates phosphatase and tensin homologue deleted on chromosome 10 through microRNA-mediated control of DNA methylation-a novel mechanism suppressing liver fibrosis. FEBS J. 2014; 281:88–103. 10.1111/febs.12574 24138392

[48] Richards KL, Zhang B, Baggerly KA, Colella S, Lang JC, Schuller DE, et al Genome-wide hypomethylation in head and neck cancer is more pronounced in HPV-negative tumors and is associated with genomic instability. PLoS One. 2009;4:e4941 10.1371/journal.pone.0004941 19293934 PMC2654169

[49] Seisenberger S, Peat JR, Hore TA, Santos F, Dean W, Reik W. Reprogramming DNA methylation in the mammalian life cycle: building and breaking epigenetic barriers. Philos Trans R Soc Lond B Biol Sci. 2013;5:368. 10.1098/rstb.2011.0330 PMC3539359 23166394

[50] Berdasco M, Esteller M. DNA methylation in stem cell renewal and multipotency. Stem Cell Res Ther. 2011;2:42 10.1186/scrt83 22041459 PMC3308039

[51] Wu H, Zhang Y. Reversing DNA methylation: mechanisms, genomics, and biological functions. Cell. 2014;156:45–68. 10.1016/j.cell.2013.12.019 24439369 PMC3938284

