# Supplementary figures and images for: Epigenetic Changes during Hepatic Stellate Cell Activation
Source: PLoS One. 2015 Jun 12;10(6):e0128745. doi: 10.1371/journal.pone.0128745 (PMC4466775; doi:10.1371/journal.pone.0128745)

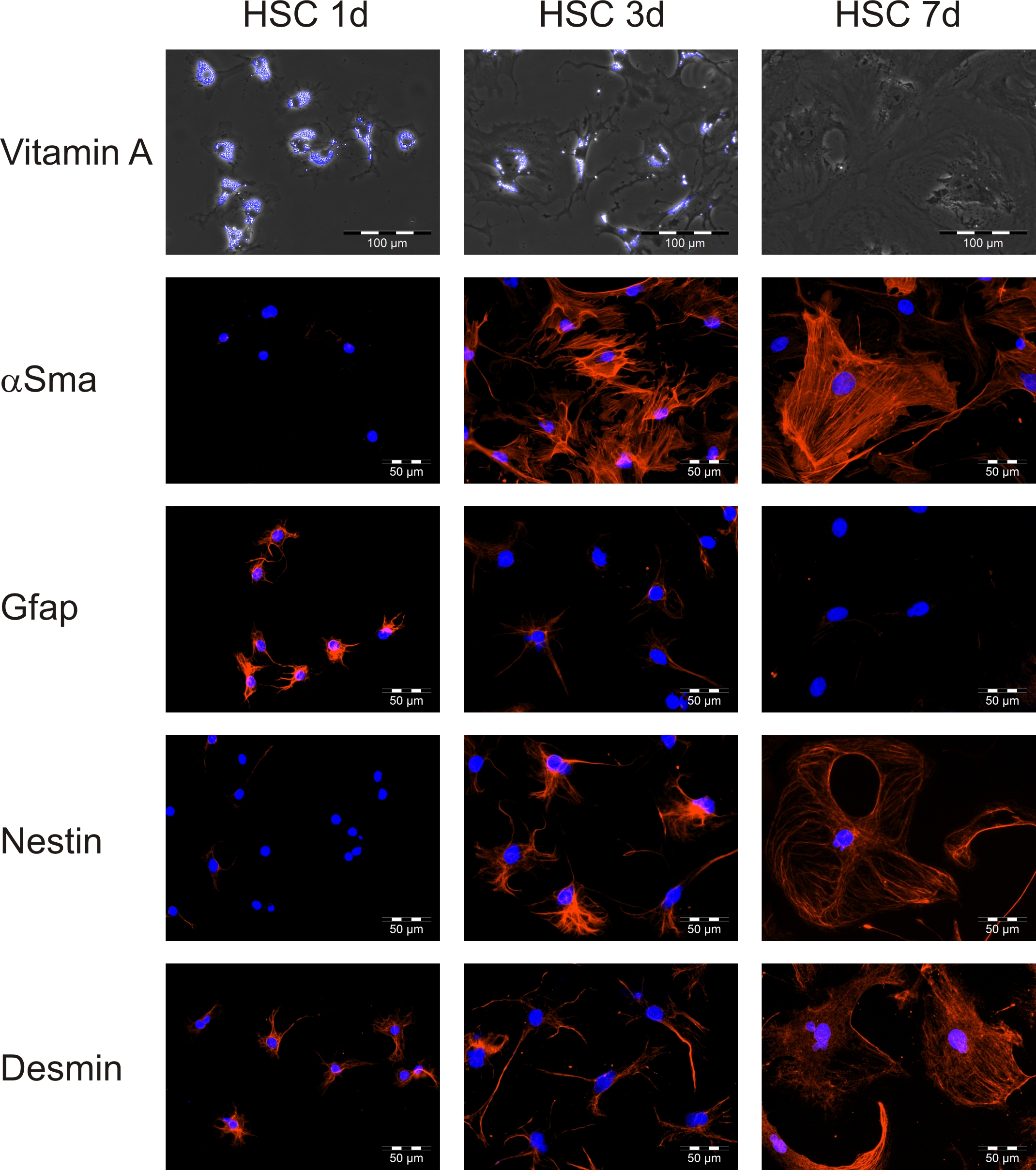

Supplement: S1 Fig — Vitamin A content of HSC decreased with culture time as displayed by the autofluorescence of vitamin A (blue). IF staining of αSma, Gfap, Nestin and Desmin (red) showed an increase in activation-associated factors αSma and Nestin during culture, while Gfap decreased. The nuclei were stained with DAPI (blue). (TIF) [file pone.0128745.s001.tif]

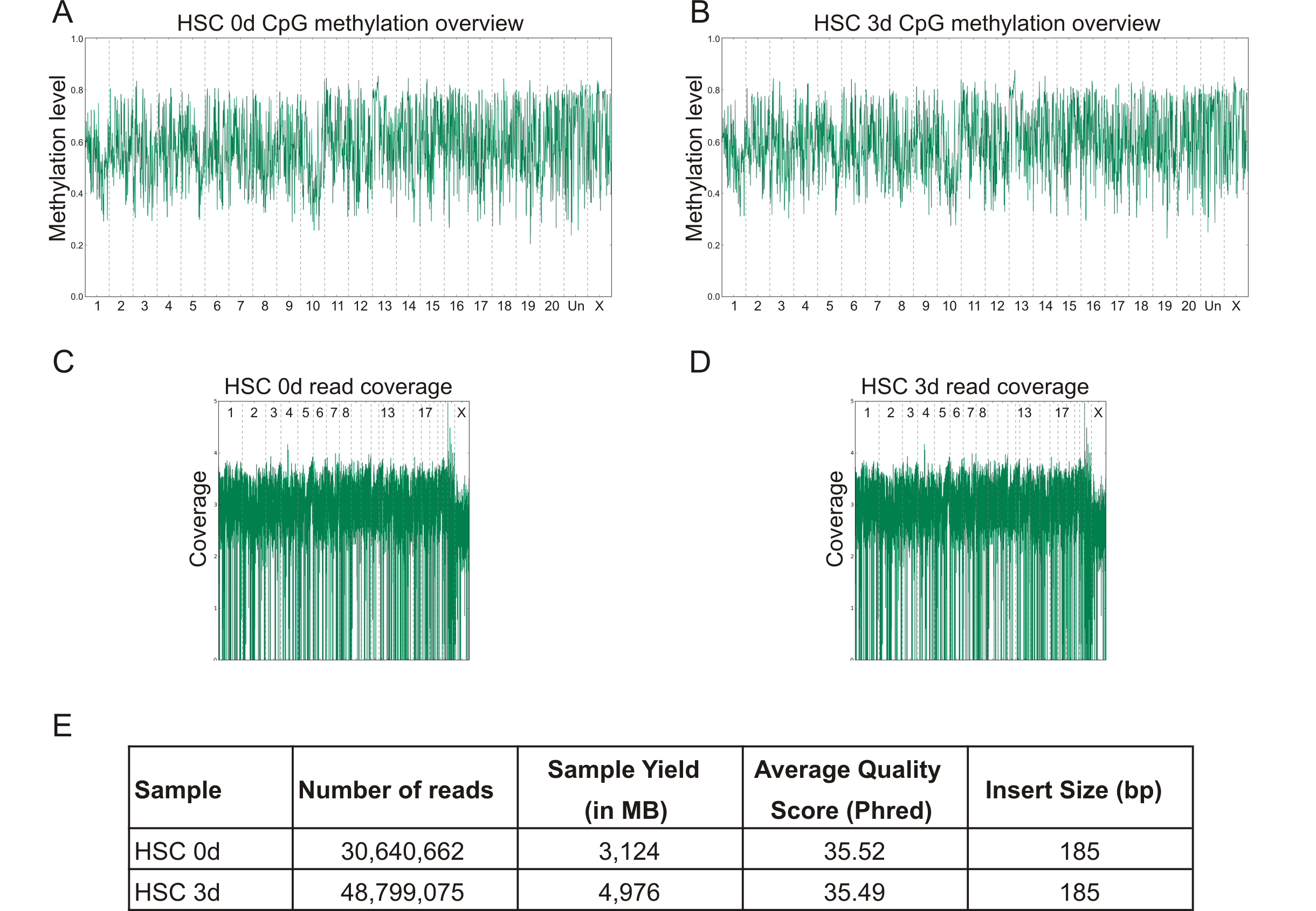

Supplement: S2 Fig — (A+B) Overview of DNA methylation measured within CpG-dinucleotides in HSC 0d and 3d throughout the genome. No significant amount of non-CpG methylation were detected throughout the genome, which indicates that the bisulfite modification and subsequent EpiQuest sequencing worked properly. (C+D) These diagrams display the coverage of the analyzed sequences at the different chromosomes for both samples showing that all chromosome were at least partially included in the analysis. (E) Table of the quality statistics of the EpiQuest sequencing. (TIF) [file pone.0128745.s002.tif]

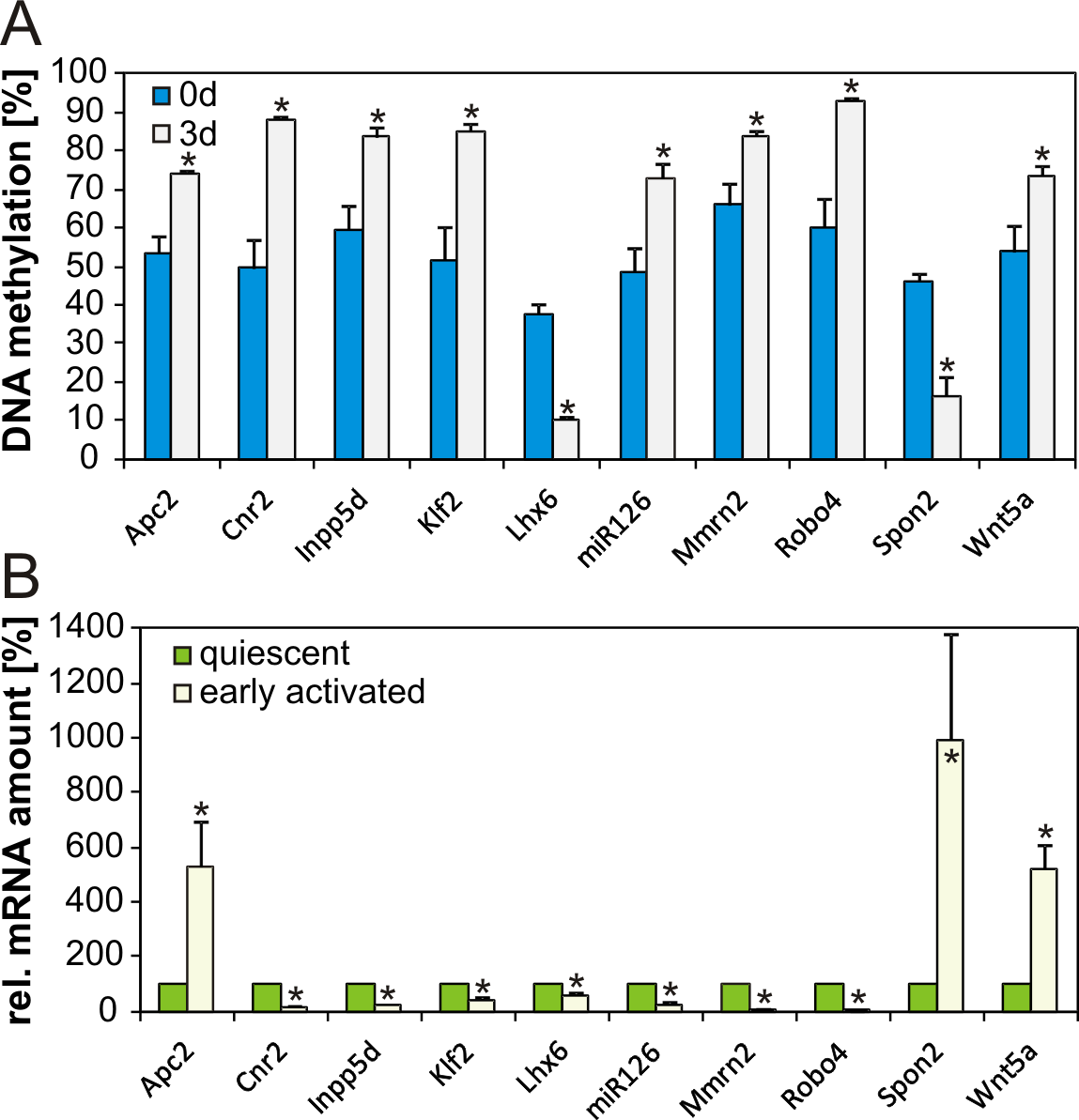

Supplement: S3 Fig — (A) DNA methylation was determined by direct bisulfite sequencing and showed significant changes of DNA methylation during in vitro activation (n = 3 independent experiments; * p<0.05). (B) The corresponding quantitative gene expression analysis was determined with quiescent (cultured overnight) and early activated (3 days cultured) HSC (n = 5 independent experiments; * p<0.05). These analyses revealed a good correlation between DNA methylation changes and gene expression, if the position of the DNA methylation was included (Table 2). (TIF) [file pone.0128745.s003.tif]

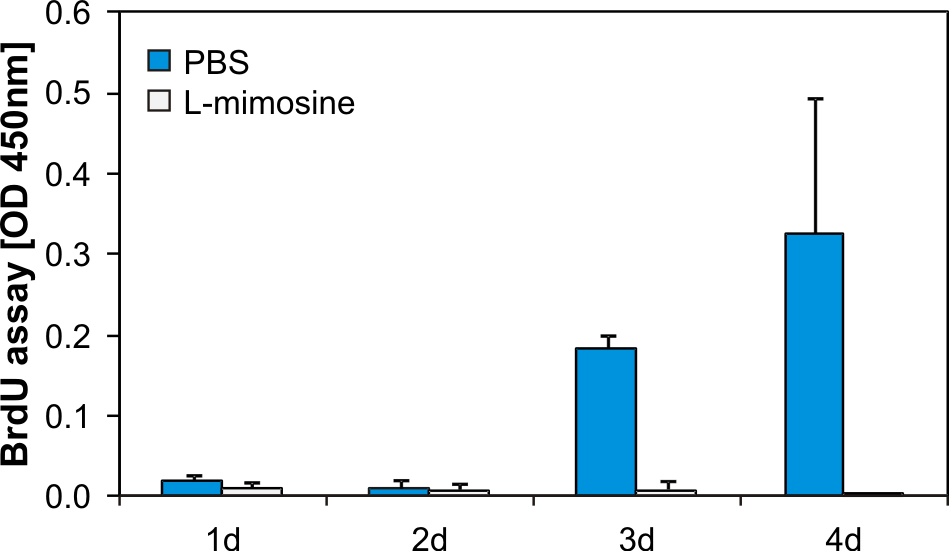

Supplement: S4 Fig — The DNA synthesis of L-mimosine or PBS control treated HSC in culture were measured with a BrdU assay (n = 3). The analysis revealed that the L-mimosine treatment almost completely blocked the DNA synthesis in cultured HSC while DNA synthesis was induced at day 3 of culture in control treated cells. (TIF) [file pone.0128745.s004.tif]
