# Supplementary material for: Epigenetic Changes during Hepatic Stellate Cell Activation
Source: PLoS One. 2015 Jun 12;10(6):e0128745. doi: 10.1371/journal.pone.0128745 (PMC4466775; doi:10.1371/journal.pone.0128745)
Supplement: S1 Table — (PDF) [file pone.0128745.s005.pdf]

Antibody	Company	Product number	Method	Dilution	Dilution buffer
Dnmt1	Cell Signaling	5032	WB	1:2,500	milk-powder
Dnmt3a	Santa Cruz	sc-20703	WB	1:2,500	milk-powder
Dnmt3b	Cayman Chemicals	13485	WB	1:5,000	milk-powder
Ki67	Chemicon	AB9260	WB	1:5,000	BSA
g-Tubulin	Sigma	T5326	WB	1:10,000	milk-powder
aSma	Dako	IR611	IF	1:200	2% FCS
Gfap	Chemicon	MAB3402	IF	1:200	2% FCS
Desmin	Abcam	ab8592	IF	1:200	2% FCS
Nestin	Santa Cruz	sc-33677	IF	1:200	2% FCS
