# Supplementary material for: Epigenetic Changes during Hepatic Stellate Cell Activation
Source: PLoS One. 2015 Jun 12;10(6):e0128745. doi: 10.1371/journal.pone.0128745 (PMC4466775; doi:10.1371/journal.pone.0128745)
Supplement: S2 Table — (PDF) [file pone.0128745.s006.pdf]

Gene Symbol	Genomic context	Range	Primer sequence fwd	Primer sequence rev	product [bp]	Annealing temp.
Apc2	NC_005106.4	12258468..12280466	TTTGGGTGGTGGGTATAGAGATA	CTTCATTAAAAAATCCCCAAATCT	374	52°C
Cnr2	NC_005104.4	54242214..154268126	GGTTTGGAGTTTAATTTTATGAAGG	ACATAACACCAAAAACCACCAATA	427	54°C
Inpp5d	NC_005108.4	94745220..94850778	GGGAGTGGTTGTTGATTTAGTTTAG	CCTAACACCACACAAACCTATCATA	413	53°C
Klf2	NC_005115.4	9223087..19225037	GGAATTGGTGGTAAAGTTATTTTTTT	ATCAACTTCAAAAACCCAAAAATC	478	53°C
Lhx6	NC_005102.4	5409760..15433504	GATTAGGAAGTTAGGTGTTAGGGAA	CCAATCAATCAACTTAATAAAATTTAA	478	53°C
Mir126	NC_005102.4	4042488..4042560	GGGTGGAGGTTAGTATTATGTTGAG	AAAAAATTTCCTATCCCACAAAAAC	430	53°C
Mmrn2	NC_005115.4	0727497..10749307	AAATTTGTTGGAAAGTTGAGGTTAG	CAACCACTCAAATCAATTCCTATATC	363	54°C
Robo4	NC_005107.4	39082701..39093341	GGAGGTAAGGTTTTGGATTTTTTAT	CAACCTAACTCTACCTCACCCTAAAC	379	56°C
Spon2	NC_005113.4	82818735..82824529	GTTTATTGTAGTTTAGTGTTAGGGAAAT	CCCAACCAAAAACTTCAAACTTCTTA	397	56°C
Wnt5a	NC_005115.4	4469451..4488707	AAGGAGAAGTTTATTTTTTGGATTGT	ATTCTCCAATCTACACTTTCTCTAAACC	395	56°C
