# Supplementary material for: Epigenetic Changes during Hepatic Stellate Cell Activation
Source: PLoS One. 2015 Jun 12;10(6):e0128745. doi: 10.1371/journal.pone.0128745 (PMC4466775; doi:10.1371/journal.pone.0128745)
Supplement: S3 Table — (PDF) [file pone.0128745.s007.pdf]

Gene Symbol	Accession No.	Primer sequence fwd	Primer sequence rev	product [bp]
Apc2	NM_001106769	GCCTGCTGGAAGAACTAGACC	CGTGTCTACGTGTGGCAGTTC	137
Cnr2	NM_001164143	GATCTCCTACCTACCGCTCAT	TTCCAGAGGACATACCCATAG	156
Inpp5d	NM_019311.1	TTCCTCTTCCAACAGAGAACC	TGCTCAGATAATCCTGGATGG	141
Klf2	NM_001007684	AGCCTATCTTGCCGTCCTTT	GTCCCATGGACAGGATGAAGT	141
Lhx6	NM_001107837.1	CCGTCTGCTGGCAAGAATATC	AGCAGCTATTCTGCTGCCTCA	145
Mir126	NR_031871.1	TGACAGCACATTATTACTTTTG	TGACCACGCATTATTACTCA	73
Mmrn2	XM_003752862.1	TCCAGGTTCTCCAACCCTAAT	CTGTTTTGCAGGCAGCTACA	148
Robo4	NM_181375.1	CCTATATGTGTATGGCCACCAA	TTTTACAGGTTCTGGGTTCAGC	158
Rps6	NM_017160	GGAAGCGCAAGTCTGTCCGA	AGGTCCCAACCGACGAGGCA	131
Spon2	NM_138533.3	CAGGTGGTCCTTGATCTTTAC	AATGAGTTGGCTGGGTGACT	140
Wnt5a	NM_022631.1	GCAGCACAGTGGACAACACT	GGCTCATGGCATTTACCACT	120
