# Supplementary material for: Epigenetic Changes during Hepatic Stellate Cell Activation
Source: PLoS One. 2015 Jun 12;10(6):e0128745. doi: 10.1371/journal.pone.0128745 (PMC4466775; doi:10.1371/journal.pone.0128745)
Supplement: S4 Table — (PDF) [file pone.0128745.s008.pdf]

Repetitive Element	Consensus RepBase	Primer sequence fwd	Primer sequence rev	product [bp]
Erv1	LTR3_Rn	CCAACATTCCTTTACCCTCT	TGATGGAACAGGATAGGAGA	121
Erv2	LTR2_Rn	CCAGACCTCCTAGATAGTGAA	GCCCACCACCTTCCTCTAAT	147
Erv3	LTR9_Rn	CCATTGTTCATACCAAGCTG	GGTCACAAGCCTTGCCTATTT	135
Ervb1	ERVB1_1-LTR_RN	TCGCTGAGCCTTTTTAGCCT	CCGACCCAATCAAGGGCATA	164
Ervb2	ERVB2_2-LTR_RN	CCGTGCCTACAGCAGAGATA	AGTCCAGGTGCACATCTTCTT	138
ID	ID_Rn1	GGCTGGGGATTTAGCTCAGT	TGGTTCTTTTTTTCGGAGCTGG	86
LINE1 (1)	L1_RN	CACTCCTGACACACAGGCTTA	CGATGATGCCATGTAGTCTTG	139
LINE1 (2)	L1_RN	CCACCCAATTAGACAAGATGG	TCTCAGTTCAGTGGTTTGCTG	135
SRV	SRV_RN_I	CAGATGAATTCCCTTGCTCAG	CAAGCCAGATTCGTTGACAT	129
Actb (A)	NM_031144	GTGATAAATGGCCTTGGAGTG	TGAGTGAGACATGCAAAGAGG	124
Actb (B)	NM_031144	CCAACTTTACCTTGGCCACT	CCCTAGGCGGAAAGTTAAGC	129
