# Supplementary material for: Epigenetic Changes during Hepatic Stellate Cell Activation
Source: PLoS One. 2015 Jun 12;10(6):e0128745. doi: 10.1371/journal.pone.0128745 (PMC4466775; doi:10.1371/journal.pone.0128745)
Supplement: S5 Table — (PDF) [file pone.0128745.s009.pdf]

S5 Table. UCSC genome browser tracks obtained from EpiQuest sequencing for rat genome browser assembly Baylor3.4/rn4.

Sample	link UCSC track
HSC 0d CHG meth.	track name='4792 CHG meth' bigDataUrl=https://s3.amazonaws.com/epiquest/epiquest_bc55925/SG0HXKEFQ67HH740I69XZEICWRP2EPYY/Analysis/browserTracks/bc55925_1_CHG_meth.bb type=bigBed itemRgb=On visibility=3
HSC 0d CHH meth.	track name='4792 CHH meth' bigDataUrl=https://s3.amazonaws.com/epiquest/epiquest_bc55925/SG0HXKEFQ67HH740I69XZEICWRP2EPYY/Analysis/browserTracks/bc55925_1_CHH_meth.bb type=bigBed itemRgb=On visibility=3
HSC 0d CpG meth.	track name='4792 CpG meth' bigDataUrl=https://s3.amazonaws.com/epiquest/epiquest_bc55925/SG0HXKEFQ67HH740I69XZEICWRP2EPYY/Analysis/browserTracks/bc55925_1_CpG_meth.bb type=bigBed itemRgb=On visibility=3
HSC 3d CHG meth.	track name='4793 CHG meth' bigDataUrl=https://s3.amazonaws.com/epiquest/epiquest_bc55925/SG0HXKEFQ67HH740I69XZEICWRP2EPYY/Analysis/browserTracks/bc55925_2_CHG_meth.bb type=bigBed itemRgb=On visibility=3
HSC 3d CHH meth.	track name='4793 CHH meth' bigDataUrl=https://s3.amazonaws.com/epiquest/epiquest_bc55925/SG0HXKEFQ67HH740I69XZEICWRP2EPYY/Analysis/browserTracks/bc55925_2_CHH_meth.bb type=bigBed itemRgb=On visibility=3
HSC 3d CpG meth.	track name='4793 CpG meth' bigDataUrl=https://s3.amazonaws.com/epiquest/epiquest_bc55925/SG0HXKEFQ67HH740I69XZEICWRP2EPYY/Analysis/browserTracks/bc55925_2_CpG_meth.bb type=bigBed itemRgb=On visibility=3

Sample	Read Tracks
HSC 0d	track name='4792 reads' bigDataUrl=https://s3.amazonaws.com/epiquest/epiquest_bc55925/SG0HXKEFQ67HH740I69XZEICWRP2EPYY/Alignment/bc55925_1.bam type=bam visibility=3
HSC 3d	track name='4793 reads' bigDataUrl=https://s3.amazonaws.com/epiquest/epiquest_bc55925/SG0HXKEFQ67HH740I69XZEICWRP2EPYY/Alignment/bc55925_2.bam type=bam visibility=3
