# Supplementary material for: Epigenetic Changes during Hepatic Stellate Cell Activation
Source: PLoS One. 2015 Jun 12;10(6):e0128745. doi: 10.1371/journal.pone.0128745 (PMC4466775; doi:10.1371/journal.pone.0128745)
Supplement: S7 Table — (PDF) [file pone.0128745.s011.pdf]

S7 Table. Complete list of GO terms generated by DAVID with differentially methylated genes during HSC activation sorted by p-value (count >5, p<0.01).

GO ID	GO Term	Count	Fold Enrich.	PValue
GO:0051239	regulation of multicellular organismal process	40	2.44	2.54E-07
GO:0065007	biological regulation	125	1.39	8.45E-07
GO:0050789	regulation of biological process	118	1.42	1.16E-06
GO:0002694	regulation of leukocyte activation	14	5.25	2.58E-06
GO:0050865	regulation of cell activation	14	5.03	4.15E-06
GO:0051249	regulation of lymphocyte activation	13	5.45	4.56E-06
GO:0002682	regulation of immune system process	20	3.20	1.54E-05
GO:0006955	immune response	21	3.06	1.69E-05
GO:0050794	regulation of cellular process	108	1.38	2.20E-05
GO:0007165	signal transduction	49	1.83	2.97E-05
GO:0006954	inflammatory response	14	3.89	6.36E-05
GO:0002683	negative regulation of immune system process	9	6.46	7.15E-05
GO:0006952	defense response	19	2.87	1.09E-04
GO:0009605	response to external stimulus	29	2.15	1.74E-04
GO:0002695	negative regulation of leukocyte activation	7	7.97	2.26E-04
GO:0009611	response to wounding	19	2.70	2.32E-04
GO:0003013	circulatory system process	11	4.28	2.47E-04
GO:0008015	blood circulation	11	4.28	2.47E-04
GO:0044057	regulation of system process	16	3.03	2.65E-04
GO:0032879	regulation of localization	24	2.31	2.68E-04
GO:0040012	regulation of locomotion	12	3.85	2.86E-04
GO:0002376	immune system process	26	2.19	3.06E-04
GO:0050866	negative regulation of cell activation	7	7.54	3.07E-04
GO:0048518	positive regulation of biological process	50	1.61	5.17E-04
GO:0050863	regulation of T cell activation	9	4.85	5.26E-04
GO:0050670	regulation of lymphocyte proliferation	8	5.48	6.17E-04
GO:0032944	regulation of mononuclear cell proliferation	8	5.42	6.60E-04
GO:0002526	acute inflammatory response	8	5.36	7.06E-04
GO:0070663	regulation of leukocyte proliferation	8	5.30	7.55E-04
GO:0001817	regulation of cytokine production	10	3.89	0.001
GO:0051241	negative regulation of multicellular organismal process	10	3.82	0.001
GO:0048519	negative regulation of biological process	43	1.63	0.001
GO:0002443	leukocyte mediated immunity	7	5.63	0.001
GO:0051250	negative regulation of lymphocyte activation	6	7.10	0.001
GO:0030334	regulation of cell migration	10	3.66	0.002
GO:0010628	positive regulation of gene expression	20	2.21	0.002
GO:0065008	regulation of biological quality	37	1.66	0.002
GO:0040013	negative regulation of locomotion	6	6.35	0.002
GO:0048731	system development	51	1.49	0.003
GO:0006909	phagocytosis	5	8.15	0.003
GO:0031328	positive regulation of cellular biosynthetic process	22	2.00	0.003
GO:0048856	anatomical structure development	53	1.46	0.003
GO:0030154	cell differentiation	39	1.60	0.003

GO ID	GO Term	Count	Fold Enrich.	PValue
GO:0032501	multicellular organismal process	92	1.28	0.003
GO:0007275	multicellular organismal development	56	1.44	0.003
GO:0048522	positive regulation of cellular process	43	1.54	0.004
GO:0051270	regulation of cell motion	10	3.24	0.004
GO:0009891	positive regulation of biosynthetic process	22	1.97	0.004
GO:0002252	immune effector process	8	3.99	0.004
GO:0010646	regulation of cell communication	28	1.77	0.004
GO:0048771	tissue remodeling	6	5.66	0.004
GO:0002460	adaptive immune response based on somatic recombination of immune receptors built from immunoglobulin superfamily domains	6	5.48	0.005
GO:0002250	adaptive immune response	6	5.48	0.005
GO:0032502	developmental process	60	1.39	0.005
GO:0046903	secretion	12	2.71	0.005
GO:0048584	positive regulation of response to stimulus	11	2.86	0.005
GO:0002684	positive regulation of immune system process	11	2.86	0.005
GO:0007242	intracellular signaling cascade	27	1.76	0.005
GO:0051251	positive regulation of lymphocyte activation	7	4.35	0.005
GO:0045935	positive regulation of nucleobase, nucleoside, nucleotide and nucleic acid metabolic process	20	1.98	0.006
GO:0009893	positive regulation of metabolic process	26	1.76	0.006
GO:0006950	response to stress	36	1.57	0.006
GO:0048869	cellular developmental process	39	1.54	0.006
GO:0048583	regulation of response to stimulus	16	2.18	0.007
GO:0007167	enzyme linked receptor protein signaling pathway	12	2.59	0.007
GO:0050801	ion homeostasis	15	2.25	0.007
GO:0001775	cell activation	11	2.74	0.007
GO:0042127	regulation of cell proliferation	21	1.90	0.007
GO:0030030	cell projection organization	14	2.34	0.007
GO:0019932	second-messenger-mediated signaling	9	3.21	0.007
GO:0045941	positive regulation of transcription	18	2.03	0.007
GO:0051173	positive regulation of nitrogen compound metabolic process	20	1.93	0.008
GO:0048523	negative regulation of cellular process	37	1.54	0.008
GO:0010557	positive regulation of macromolecule biosynthetic process	20	1.92	0.008
GO:0002696	positive regulation of leukocyte activation	7	3.98	0.008
GO:0051240	positive regulation of multicellular organismal process	11	2.66	0.008
GO:0006873	cellular ion homeostasis	14	2.28	0.008
GO:0055082	cellular chemical homeostasis	14	2.25	0.009
GO:0010604	positive regulation of macromolecule metabolic process	24	1.75	0.009
GO:0050867	positive regulation of cell activation	7	3.84	0.010
GO:0003018	vascular process in circulatory system	5	5.91	0.010
GO:0007187	G-protein signaling, coupled to cyclic nucleotide second messenger	6	4.52	0.010
